# Comparison of high-intensity interval training and small-sided games on physical fitness among players: a systematic review with meta-analysis

**DOI:** 10.3389/fphys.2026.1754825

**Published:** 2026-03-31

**Authors:** Yu Zeng, Xinzhi Wang, Kim Geok Soh, Shuzhen Ma, Yue Zhao, Wenchao Rong, Xinrui Zhang, Ningfei Wei

**Affiliations:** 1 College of Physical Education and Health, Guangxi Normal University, Guilin, China; 2 Faculty of Sports Studies, Shandong University of Technology, Zibo, China; 3 Faculty of Educational Studies, University Putra Malaysia, Serdang, Malaysia; 4 School of Public Administration, Guilin University of Technology, Guilin, China; 5 School of Physical Education, Shandong Sport University, Shandong, China; 6 College of Physical and Health Sciences, Guangxi Minzu University, Nanning, China

**Keywords:** high-intensity interval training, physical fitness, players, small-sided games, sprint interval training

## Abstract

**Background:**

With the continuous advancement of sports training methodologies, high-intensity interval training (HIIT) and small-sided games (SSG) have emerged as crucial strategies for enhancing players’ physical fitness. However, the comparative effects of these training modalities on various fitness components and their underlying physiological adaptation mechanisms remain insufficiently explored.

**Objectives:**

This systematic review and meta-analysis aim to compare the overall impact of HIIT and SSG on players’ physical fitness and examine the moderating effects of different training parameters, including training frequency, intervention duration, players’ training experience, age, and gender. The findings seek to provide theoretical insights for optimising training protocols.

**Methods:**

A systematic search was conducted in databases including PubMed, Web of Science, Scopus, and EBSCOhost to identify relevant English-language randomised controlled trials that met predefined eligibility criteria. Hedges’ g was used as the effect size metric, and a random-effects model was applied for meta-analyses of power, speed, endurance, and agility. Subgroup analyses were performed to assess the influence of potential moderators.

**Results:**

The meta-analysis revealed that, compared to SSG, HIIT produced a small but statistically significant improvement in speed (ES = 0.564) and a moderate and statistically significant improvement in endurance (ES = 0.706). In contrast, gains in power (ES = −0.144) and agility (ES = −0.159) were trivial in magnitude and not statistically significant. Subgroup analyses indicated that lower training frequency (<3 sessions per week) and shorter intervention duration (<6 weeks) yielded significant improvements. Furthermore, players across different age groups benefited from HIIT, while sex did not exhibit a significant moderating effect.

**Conclusion:**

This systematic review and meta-analysis confirm that both HIIT and SSG effectively enhance players’ physical fitness, with HIIT demonstrating a distinct advantage in improving speed and endurance. Although HIIT showed relatively limited improvements in power and agility compared to SSG, both training modalities exert their effects through distinct physiological adaptation mechanisms. Subgroup analysis findings suggest that appropriately balancing training frequency and intervention duration is crucial for achieving optimal training outcomes. Future research should further investigate the long-term effects of these interventions to provide a more robust theoretical foundation for personalised training program design.

**Systematic Review Registration:**

https://www.crd.york.ac.uk/prospero/, identifier CRD42024555633.

## 1 Introduction

Physical fitness serves as the foundational pillar of athletic performance, encompassing attributes such as endurance, strength, speed, agility, and power (Hoffman et al., 2019). These elements collectively enable players to meet the physical and physiological demands of their respective sports. Therefore, selecting effective training methodologies is crucial for optimizing performance and reducing injury risk. Among the myriad training strategies employed in sports, small-sided games (SSG) and high-intensity interval training (HIIT) have garnered significant attention for their effectiveness in enhancing physical fitness (Arslan et al., 2022). However, a systematic comparison of these two approaches and their impact on players’ fitness levels remains an underexplored area. SSG is a training modality grounded in sport-specific simulations, involving modified versions of team sports with reduced player numbers, smaller field dimensions, and adjusted rules (Paprancova et al., 2024). Designed to replicate the technical, tactical, and physical demands of competitive play, SSG enables targeted training objectives. Its contextual nature not only improves physical attributes such as aerobic capacity, speed, and agility but also fosters decision-making skills and game awareness (Sarmento et al., 2018). SSG has been shown to enhance cardiovascular endurance, agility, and muscular strength (Mitrotasios et al., 2021). The dual benefits of skill acquisition and fitness improvement make SSG particularly appealing to coaches and players.

In contrast, HIIT involves alternating between bouts of maximal or near-maximal intensity exercise and periods of rest or low-intensity recovery. HIIT has been extensively studied for its time-efficient approach to improving both aerobic and anaerobic fitness (Bartel et al., 2022). It induces significant physiological adaptations, such as enhanced cardiovascular function, improved metabolic efficiency, and heightened neuromuscular coordination, making it a preferred choice for players across various sports (Callahan et al., 2021). Additionally, HIIT protocols are highly customizable, allowing coaches to tailor sessions to meet the specific fitness demands of individual players or teams (Coates et al., 2023). While both SSG and HIIT have demonstrated effectiveness in improving physical fitness, they differ substantially in their underlying mechanisms, practical applications, and contextual relevance. SSG integrates physical conditioning with sport-specific skill development, providing a holistic training experience aligned with competitive scenarios (Gusic et al., 2021). Conversely, HIIT focuses on maximizing physiological adaptations in controlled and structured settings, offering versatility across different sporting contexts (Kızılet, 2021). These distinct approaches raise pertinent questions about their relative effectiveness in enhancing players’ fitness.

Previous studies have examined the individual effects of SSG and HIIT on various fitness components (Faude et al., 2014; Selmi et al., 2020; Zeng et al., 2022; Thomakos et al., 2023). However, the lack of direct comparisons between these training modalities has precluded definitive conclusions about their comparative advantages. Furthermore, differences in study designs, participant characteristics, and training protocols complicate evidence synthesis, highlighting the need for systematic meta-analysis evaluation.

This systematic review aims to address this gap by comparing the effects of SSG and HIIT on the physical fitness of players across different sports. By analysing collective evidence, this study seeks to answer the critical question: which training method SSG or HIIT is more effective in enhancing players’ physical fitness? The findings of this review will provide valuable insights for coaches, sports scientists, and players, facilitating evidence-based training program development to optimize performance on the field.

## 2 Methods

### 2.1 Protocol and registration

This systematic review and meta-analysis adhered to the Preferred Reporting Items for Systematic Reviews and Meta-Analyses (PRISMA) guidelines (Page et al., 2021). The study protocol was registered with the International Prospective Register of Systematic Reviews (PROSPERO) under registration number CRD42024555633.

### 2.2 Search strategy

A comprehensive literature search was conducted across four electronic databases: Web of Science, PubMed, Scopus, and EBSCOhost before December 2024. The search strategy incorporated relevant Medical Subject Headings (MeSH) terms and keywords to identify studies comparing the effects of SSG and HIIT on physical fitness among players. The main keywords of Boolean search strategy are (“sprint interval training” OR “high-intensity interval training” OR “Extreme Conditioning Program” OR “vigorous physical exercise” OR “high-intensity functional training”) AND (SSG OR “small-sided games” OR “drill-based” OR “constrained games” OR “sided-game”) AND (“physical fitness” OR “physical endurance” OR “cardiorespiratory fitness” OR “aerobic endurance” OR “endurance” OR “strength” OR “speed” OR “power” OR “agility” OR “balance” OR “coordination” OR “flexibility” OR “reaction time”). The search was limited to peer-reviewed articles published in English. References of eligible studies and related systematic reviews were screened to identify additional relevant articles.

### 2.3 Eligibility criteria

This systematic review and meta-analysis applied the PICOS framework as the standard for determining inclusion and exclusion criteria, following established guidelines (Amir-Behghadami and Janati, 2020). The inclusion criteria for eligible studies were defined as follows: (1) Participants must be healthy players involved in either team or individual sports, irrespective of gender; (2) The training interventions using SSG or HIIT; (3) Studies must either directly compare the effects of SSG and HIIT or compare these interventions against a control group; (4) The study must report at least one measurement of physical fitness outcomes; And (5) The included studies must be randomized controlled trials.

The exclusion criteria for eligible studies were defined as follows: (1) Studies involving players with pre-existing medical conditions, injuries, or other health issues that could interfere with physical performance; (2) Studies investigating other types of training interventions or combining SSG or HIIT with additional modalities without isolating their effects; (3) Studies that did not compare SSG with HIIT or did not involve a control group; (4) Studies that did not measure or report any physical fitness outcomes; (5) Lack of data to calculate the effect size; And (6) Abstract, non-randomized trials, observational studies, cohort studies, case studies, oral presentation, reviews, study protocol.

### 2.4 Study selection

All identified studies were imported into EndNote X9 for duplicate removal. Titles and abstracts were screened independently by two reviewers (XW and SM) based on predefined eligibility criteria. Studies deemed potentially relevant proceeded to full-text screening. Discrepancies between the reviewers were resolved by consensus or by consulting a third reviewer (KGS). The study selection process was documented using the PRISMA flow diagram.

### 2.5 Data extraction

Data extraction was performed independently by three reviewers (XW, SM, and KGS) using a pre-designed data extraction sheet. Extracted information included authors, publication year, study design, sample characteristics (age, gender, sport), intervention details (frequency, duration, and length), comparator details, and reported outcomes. The extracted data were cross-verified for accuracy. Any disagreements were resolved by discussion among the reviewers.

### 2.6 Study quality assessment

The quality of the included studies was assessed using the revised Cochrane Risk of Bias tool (ROB 2), which is designed to evaluate the risk of bias in randomized controlled trials (RCTs) (Sterne et al., 2019). ROB 2 examines five domains of bias: bias arising from the randomization process, bias due to deviations from intended interventions, bias due to missing outcome data, bias in the measurement of the outcome, and bias in the selection of the reported result. Each domain was rated as having a low risk of bias, some concerns, or a high risk of bias according to ROB 2 guidelines. Studies with a low risk of bias across all domains were classified as high-quality, whereas those with one or more domains rated as “some concerns” or “high risk” were considered to have potential bias. Any disagreements in the risk of bias assessment were resolved through discussion or consultation with a third reviewer.

### 2.7 Certainty of evidence

The certainty of evidence for each outcome was assessed using the GRADE (Grading of Recommendations, Assessment, Development, and Evaluations) framework. GRADE evaluates the certainty of evidence as very low, low, moderate, or high. The overall certainty of evidence was summarized in a GRADE evidence profile table, providing a transparent evaluation of the confidence in the findings. Any discrepancies in evidence grading were resolved through discussion among the reviewers.

### 2.8 Data synthesis and statistical analysis

Meta-analysis was conducted using Comprehensive Meta-Analysis (CMA) software (Version 3.0, Biostat Inc.). To qualify for meta-analysis, at least two studies reporting comparable data were required (Henderson et al., 2010). The mean and standard deviation of the pre- and post-intervention results were extracted from the articles. In cases where relevant data were unavailable in the articles, efforts were made to contact the corresponding authors for clarification. If the required data could not be retrieved, the study was excluded from the meta-analysis. A random-effects model, using the inverse variance method, was employed to calculate effect sizes (Hedge’s g) and 95% confidence intervals (CI) for continuous outcomes (Borenstein et al., 2010). This model accounts for variations among studies, allowing for a more generalized interpretation of the results. The interpretation of effect sizes was based on Hopkins et al. (Hopkins et al., 2009), as follows: Trivial effect size: <0.2, small effect size: 0.2–0.6, moderate effect size: 0.6–1.2, large effect size: 1.2–2.0, very large effect size: 2.0–4.0, and extremely large effect size: >4.0. Heterogeneity among studies was assessed using the I^2^ statistic, which quantifies the percentage of variability across studies due to heterogeneity rather than chance. Thresholds for interpreting I^2^ were as follows: Low heterogeneity: ≤25%, moderate heterogeneity: 26%–75%, High heterogeneity: >75% (Julian et al., 2023). To evaluate publication bias, funnel plots were visually inspected, and the Egger regression test was performed to detect potential asymmetry (Lin et al., 2021). A sensitivity analysis was conducted to examine the robustness of the findings by systematically excluding individual studies and assessing the impact on overall results.

### 2.9 Additional analysis

Subgroup analyses were conducted to explore the potential moderating effects of various factors on the outcomes. Participants were classified based on the median intervention duration (≤6 weeks vs. >6 weeks) and training frequency (≤3 sessions vs. >3 sessions per week). Median values were calculated if at least three studies provided relevant data for each moderating factor. Additional moderators considered included gender (male vs. female) and age (≥16 years vs. <16 years). These analyses aimed to determine whether the effects of SSG and HIIT on physical fitness varied across specific contexts or populations.

## 3 Results

### 3.1 Study selection

**FIGURE 1:**
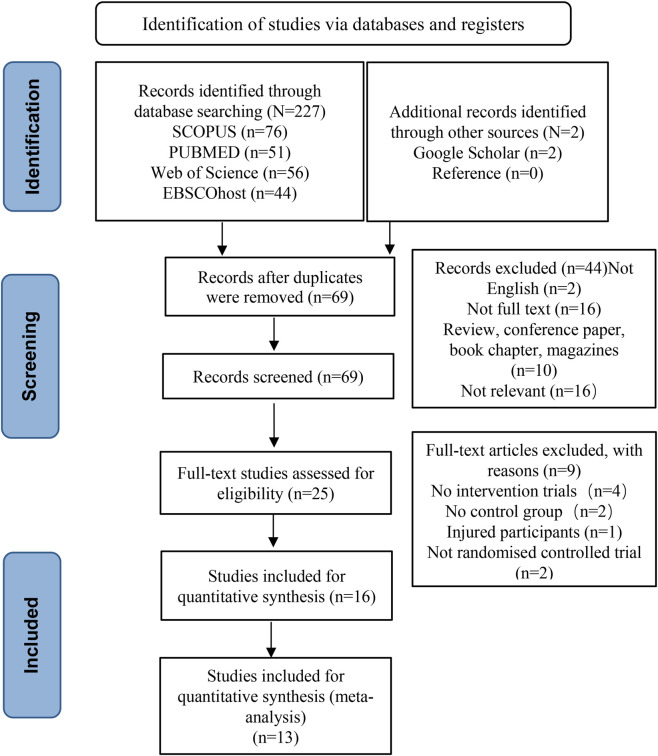
Flow chart of PRISMA article screening.

Figure 1 shows the article selection process for this systematic review. Initially, 227 articles were discovered through database search. In addition, two articles were discovered through Google Scholar and the reference list. After manually removing duplicates through EndNote software, 69 articles were retained. Then, by filtering the titles and abstracts, it was found that 25 articles were identified as meeting the criteria for full-text analysis. However, after conducting a full-text screening, it was found that nine articles were excluded. Ultimately, 13 articles met all inclusion criteria for this systematic review and meta-analysis.

### 3.2 Study quality assessment

**FIGURE 2:**
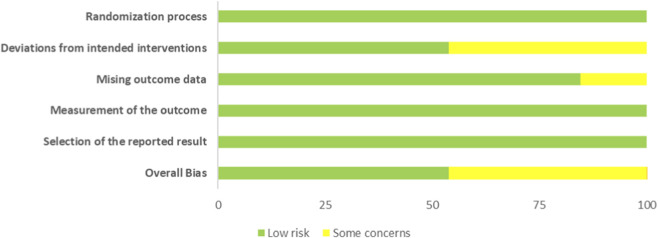
RoB-2 assessments.

**FIGURE 3:**
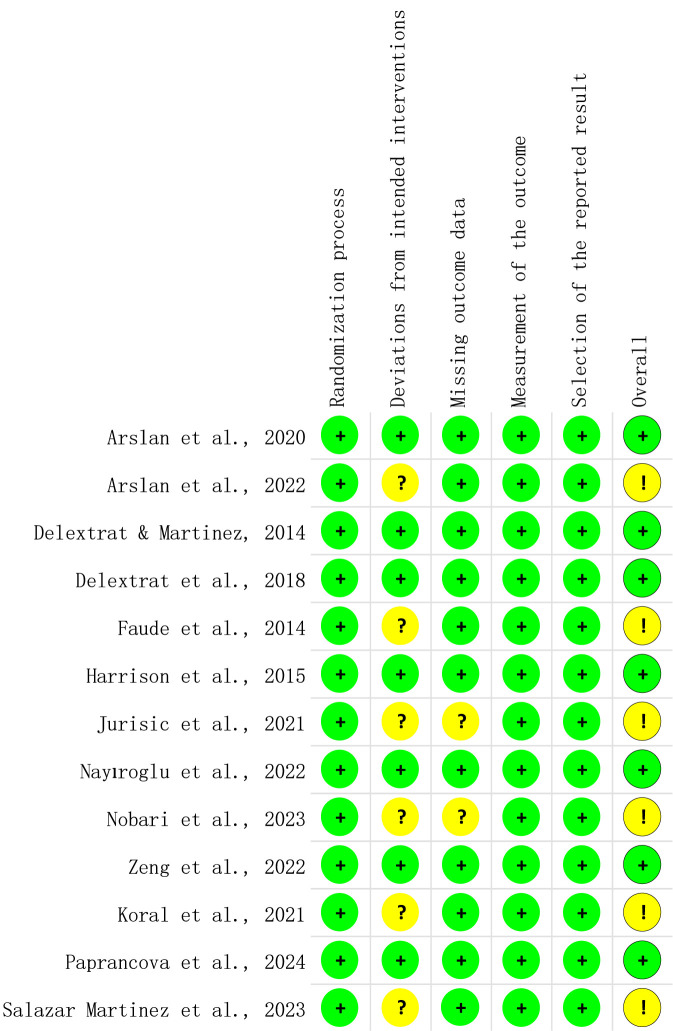
Risk of overall bias.

Figure 2 presents a detailed risk of bias assessment conducted using the ROB 2 tool, while Figure 3 reports the overall risk of bias. The findings indicate a low risk of bias in areas such as the randomisation process, outcome measurement, and selective reporting. However, some studies exhibited a degree of bias related to deviations from the intended interventions and missing outcome data. Overall, despite the generally low risk of bias, these methodological flaws need to be addressed to further improve the credibility and reliability of the research.

### 3.3 Certainty of evidence

Table 1 shows the results of GRADE analysis. The GRADE evaluation results report a low level of certainty in the evidence.

**TABLE 1:** Certainty of evidence for meta-analysed outcomes.

Outcomes	Certainty assessment					No of participants and studies	Certainty of the evidence (GRADE)
	Risk of bias	Inconsistency	Indirectness	Imprecision	Risk of publication bias		
Power performance assessed follow-up: range 4–8 weeks	Serious^a^	Not serious	Not serious	Serious^b^	Not serious	205 (9RCTs)	⨁⨁◯◯ LOW
Speed performance assessed follow-up: range 4–8 weeks	Serious^a^	Not serious	Not serious	Serious^b^	Not serious	269 (11RCTs)	⨁⨁◯◯ LOW
Endurance performance assessed follow-up: range 3–9 weeks	Serious^a^	Not serious	Not serious	Serious^b^	Not serious	219 (8RCTs)	⨁⨁◯◯ LOW
Agility performance assessed follow-up: range 3–9 weeks	Serious^a^	Not serious	Not serious	Serious^b^	Not serious	270 (10RCTs)	⨁⨁◯◯ LOW

GRADE, grading of recommendations assessment, development and evaluation; RCT, randomized controlled trial.

^a^ Some included articles had some concerns of bias.

^b^ Downgraded by one level, as ≥ 400 participants were available for a comparison but there was an unclear direction of the effects. Downgraded by two levels in case of imprecision based on both assessed points.

### 3.4 Participant characteristics

Table 2 summarises the characteristics of participants in the included studies as follows: (1) Publication Year: The studies were published between 2014 and 2024. (2) Sample Size: A total of 335 participants were included across 13 studies, with sample sizes ranging from 18 to 50. (3) Age: Participants were aged between 14 and 24 years (4) Gender: Of the 13 studies, four focused on female participants (Paprancova et al., 2024; Zeng et al., 2022; Jurisic et al., 2021; Nayıroglu et al., 2022), seven on male participants (Arslan et al., 2022; Faude et al., 2014; Arslan et al., 2020; Delextrat and Martinez, 2014; Delextrat et al., 2018; Harrison et al., 2015; Salazar Martinez et al., 2023), and two did not report gender (Koral et al., 2021; Nobari et al., 2023). (5) Training Programs: Seven studies involved soccer players (Paprancova et al., 2024; Faude et al., 2014; Nayıroglu et al., 2022; Arslan et al., 2020; Salazar Martinez et al., 2023; Koral et al., 2021; Nobari et al., 2023), four focused on basketball players (Arslan et al., 2022; Zeng et al., 2022; Delextrat and Martinez, 2014; Delextrat et al., 2018), one examined hockey players (Harrison et al., 2015), and one investigated handball players (Jurisic et al., 2021).

**TABLE 2:** Summary of the studies’ characteristics included in this review.

Study	Participants characteristics					Intervention	Comparator	Outcomes
	n	Sex	Age	Sport	Exp			
Arslan et al. (2020)	20	20M	14.2 ± 0.5 years	soccer players	3.4 ± 0.3 years	Freq.: 2 times/week time: 90 min length: 5 weeks	EG: Hight-intensity interval training CG: Small-sided game training	CMJ↓ SJ↓ 30 m Sprint↑ 1,000 m↑ RSA total↑ ZAB↓
Arslan et al. (2022)	32	32M	14.5 ± 0.5 years	basketball players	at least 3 years	Freq.: 3 times/week time: NR length: 6 weeks	EG: Hight-intensity interval training CG: Small-sided game training	CMJ↓ SJ↓ 30 m Sprint↑ RSA total↑ T-Drill↓
Delextrat and Martinez (2014)	18	18M	16.0 ± 0.6 years	basketball players	6.8 ± 3.1 years	Freq.: 2 times/week time: 26 min length: 6 weeks	EG: Hight-intensity interval training CG: Small-sided game training	3MBCP↓ Horizontal jump↓ RSA test↑ Agility T-test↓
Delextrat et al. (2018)	20	20M	14.3 ± 0.6 years	basketball players	5.2 ± 3.6 years	Freq.: 2 times/week time: NR length: 6 weeks	EG: Hight-intensity interval training CG: Small-sided game training	Sprints test↑ Total distance covered↑
Faude et al. (2014)	19	19M	16.5 ± 0.8 years	soccer players	NR	Freq.: 4 times/week time: NR length: 4 weeks	EG: Hight-intensity interval training group CG: Small-sided game training	CMJ↓ 30 m sprint time↑ COD↑
Harrison et al. (2015)	21	21M	13.9 ± 0.3 years	field hockey and rugby players	NR	Freq.: 2 times/week time: 24 min length: 6 weeks	EG: Mixed high-intensity intermittent running and SSG training CG: Small-sided game training	CMJ↑ 20 m sprint time↑
Jurisic et al. (2021)	24	24F	16.06 ± 0.80 years	handball players	at least 6 years	Freq.: 2 times/week time: 90 min length: 8 weeks	EG: Hight-intensity interval training CG: Small-sided handball game	CMJ↓ SJ↓ 30 m sprint time↑
Nayıroglu et al. (2022)	24	24F		soccer players	5.6 ± 2.3 years	Freq.: 3 times/week time: NR length: 8 weeks	EG: Hight-intensity interval training CG: Small-sided game training	CMJ↓ COD↓ 30 m sprint time↑
Nobari et al. (2023)	28	NR	17.2 ± 0.4 years	soccer players	5.9 ± 1.4 years	Freq.: 2 times/week time: 90 min length: 4 weeks	EG: Small-sided game with short high-intensity interval training CG: Small-sided game with repeated sprint training	Sargent jump test↓ SLJ↓ COD↓ 30 m Sprint↑
Zeng et al. (2022)	19	19F	19.9 ± 1.1 years	basketball players	5.5 ± 1.9 years	Freq.: 3 times/week time: NR length: 8 weeks	EG: High-intensity interval training with changes of direction CG: Small-sided games	CMJ↓ COD↓ 20 m sprint time↑ RSA best↑
Koral et al. (2021)	50	NR	19.3 ± 5.1 years	soccer players	NR	Freq.: 2 times/week time: 37 min length: 3 weeks	EG: Sprint interval training CG: Small-sided game training	10 m Sprint↑ COD↑ CRMFT↑
Paprancova et al. (2024)	36	36F	15.7 ± 1.7 years	soccer players	NR	Freq.: 2 times/week time: 20 min length: 6 weeks	EG: Hight-intensity interval training group CG: Small-sided game training	30 m Sprint↑ COD↓ 30-15IFT↑
Salazar Martinez et al. (2023)	24	24M	19 ± 1 years	soccer players	11.3 ± 2.2 years	Freq.: 3 times/week time: 30 min length: 9 weeks	EG: Hight-intensity interval training CG: Small-sided game training	RSA↓ Agility ability↑

M, male; F, female; NR, no record; EG, experiment group; CG, control group; Freq, frequency; CMJ, countermovement jump; SJ, squat jump; SLJ, standing long jump; RSA, repeated sprint ability; ZAB, zigzag agility the ball; 3MBCP, a 3-kg medicine ball 2-handed chest pass; CRMFT, continuous running multistage field test; COD, change-of-direction; IFT, intermittent fitness test.

### 3.5 Intervention characteristics

Table 2 presents the intervention characteristics of the included studies as follows: (1) Intervention Type: HIIT was the primary intervention method in all studies. (2) Intervention Frequency: Among the 13 studies, eight implemented HIIT twice per week (Paprancova et al., 2024; Jurisic et al., 2021; Arslan et al., 2020; Delextrat and Martinez, 2014; Delextrat et al., 2018; Harrison et al., 2015; Koral et al., 2021; Nobari et al., 2023), four conducted it three times per week (Arslan et al., 2022; Zeng et al., 2022; Nayıroglu et al., 2022; Salazar Martinez et al., 2023), and one study reported four times per week (Faude et al., 2014). (3) Intervention duration per Session: Three studies reported a session duration of 90 min (Jurisic et al., 2021; Arslan et al., 2020; Nobari et al., 2023), while others varied—one study lasted 37 min (Koral et al., 2021), one lasted 30 min (Salazar Martinez et al., 2023), one lasted 26 min (Delextrat and Martinez, 2014), one lasted 24 min (Harrison et al., 2015), and one lasted 20 min (Paprancova et al., 2024). Five studies did not report session duration. (4) Intervention Period: Five studies lasted for 6 weeks, three lasted for 8 weeks, two lasted for 4 weeks, one lasted for 3 weeks, one lasted for 5 weeks, and one lasted for 9 weeks.

### 3.6 Outcome

#### 3.6.1 Effect of high-intensity interval training and small-sided games on power

**FIGURE 4:**
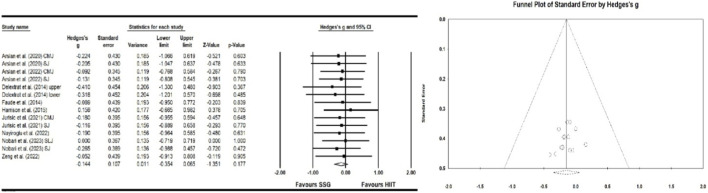
Forest plot and funnel plot of the impact of HIIT and SSG on power of players. The displayed values represent the effect size (Hedges’ g) of 95% confidence intervals (Cl). The size of the square shown in the figure represents the statistical weight.

Eight studies examined the effects of HIIT and SSG on power. The results indicate a significant improvement in power (ES = −0.144; 95% CI = −0.354–0.065; p > 0.05; Egger test p = 0.374; N = 205; Figure 4). The overall heterogeneity of the effect is low (Q = 1.435; I^2^ = 0.000%). The relative weight range for each study was 5.54%–9.62%.

#### 3.6.2 Effect of high-intensity interval training and small-sided games on speed

**FIGURE 5:**
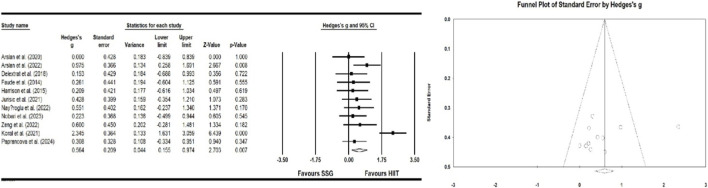
Forest plot and funnel plot of the impact of HIIT and SSG on speed of players. The displayed values represent the effect size (Hedges’ g) of 95% confidence intervals (CI). The size of the square shown in the figure represents the statistical weight.

Five studies assessed the impact of HIIT and SSG on speed performance. The results of this meta-analysis indicate that SSG and HIIT significantly improve speed (ES = 0.564; 95% CI = 0.155–0.974; p = 0.007; Egger test p = 0.335; N = 269; Figure 5). The overall heterogeneity of the effect is low (Q = 9.217; I^2^ = 0.000%). The relative weight range for each study was 8.47%–10.19%.

#### 3.6.3 Effect of high-intensity interval training and small-sided games on endurance

**FIGURE 6:**
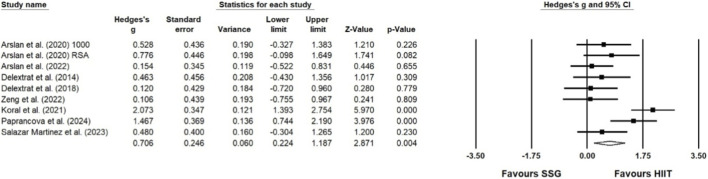
Forest plot of the impact of HIIT and SSG on endurance of players. The displayed values represent the effect size (Hedges’ g) of 95% confidence intervals (Cl). The size of the square shown in the figure represents the statistical weight.

Five studies assessed the impact of HIIT and SSG on endurance performance. The results of this meta-analysis indicate that SSG and HIIT significantly improve endurance (ES = 0.706; 95% CI = 0.224–1.187; p = 0.004; Egger test p = 0.175; N = 219; Figure 6). The overall heterogeneity of the effect is low (Q = 6.993; I^2^ = 0.000%). The relative weight range for each study was 10.32%–12.15%.

#### 3.6.4 Effect of high-intensity interval training and small-sided games on agility

**FIGURE 7:**
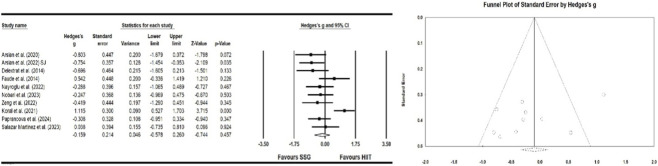
Forest plot and funnel plot of the impact of HIIT and SSG on agility of players. The displayed values represent the effect size (Hedges’ g) of 95% confidence intervals (Cl). The size of the square shown in the figure represents the statistical weight.

Six studies investigated the effects of HIIT and SSG on agility. This meta-analysis indicates a significant improvement in agility following SSG and HIIT interventions (ES = −0.159; 95% CI = −0.578–0.260; p > 0.05; Egger test p = 0.129; N = 270; Figure 7). The overall heterogeneity of the effect is low (Q = 7.651; I^2^ = 0.000%). The relative weight range for each study was 8.84%–11.65%.

#### 3.6.5 Effect of moderator variables

Table 3 shows the analysis results of mediating variables. The subgroups analysed in this study include intervention frequency (<3 times/week and ≥3 times/week), intervention length (<6 weeks and ≥6 weeks), gender (male, female, and mixed), and age (<16 years and ≥16 years). For intervention frequency, the effect size was small for studies with <3 sessions per week (ES = 0.312, 95% CI: 0.168–0.455, p < 0.001), indicating a moderate improvement in physical fitness. However, studies with ≥3 sessions per week showed an insignificant effect (ES = 0.059, 95% CI: −0.142–0.260, p = 0.566), suggesting that higher training frequency does not necessarily lead to better outcomes. The Q statistic for subgroup differences was statistically significant (p < 0.001), implying that training frequency plays a moderating role in the effectiveness of the interventions. For intervention length, studies with <6 weeks of intervention, the effect size was small to moderate (ES = 0.416, 95% CI: 0.234–0.599, p < 0.001), suggesting meaningful improvements in fitness outcomes. However, studies with ≥6 weeks showed a negligible effect (ES = 0.099, 95% CI: −0.062–0.260, p = 0.229), indicating that extending the intervention duration beyond 6 weeks may not yield additional benefits. The Q statistic for subgroup differences was significant (p < 0.001), reinforcing the influence of training duration on intervention effectiveness. For gender, studies focused exclusively on male participants showed an insignificant effect (ES = 0.137, 95% CI: −0.089–0.363, p = 0.235), as did those focusing exclusively on female participants (ES = 0.039, 95% CI: −0.239–0.317, p = 0.783). Interestingly, studies that included both male and female participants reported a moderate effect size (ES = 0.747, 95% CI: −0.085–0.578, p = 0.079), although this result was not statistically significant. The Q statistic for gender-based differences was non-significant (p = 0.451), suggesting that gender does not substantially moderate the intervention effects. For age, studies focusing on players <16 years old showed trivial effect size (ES = 0.129, 95% CI: −0.050-0.307, p = 0.158), while studies focusing on players ≥16 years old showed small effect size (ES = 0.226, 95% CI: 0.145–0.453, p < 0.001). The Q statistic of subgroup analysis showed a significant difference in intervention frequency, intervention length, and age (p < 0.001). Further subgroup analysis showed no significant difference in Q-statistics between genders (P = 0.451).

**TABLE 3:** Mediation analysis of the impact of HIIT and SSG on physical fitness.

Subgroup	Studies	ES (95%CI)	P value	Q-statistics
Intervention frequency (time/week)				
<3	29	0.312 (0.168–0.455)	<0.001^a^	
≥3	15	0.059 (−0.142 to 0.260)	0.566	
Overall	44	0.226 (0.110–0.343)	<0.001^a^	(Q = 131.480; df (Q) = 43; P < 0.001^b^)
Intervention length (week)				
<6	20	0.416 (0.234–0.599)	<0.001^a^	
≥6	24	0.099 (−0.062 to 0.260)	0.229	
Overall	44	0.238 (0.117–0.358)	<0.001^a^	(Q = 131.047; df (Q) = 43; P < 0.001^b^)
Gender				
Male	20	0.137 (−0.089 to 0.363)	0.235	
Female	17	0.039 (−0.239 to 0.317)	0.783	
Male & Female	7	0.747 (−0.085 to 0.578)	0.079	
Overall	44	0.117 (−0.047 to 0.280)	0.162	(Q = 38.135; df (Q) = 42; P = 0.451)
Age (year)				
<16	18	0.129 (−0.050 to 0.307)	0.158	
≥16	26	0.299 (0.145–0.453)	<0.001^a^	
Overall	44	0.226 (0.110–0.343)	<0.001^a^	(Q = 131.480; df (Q) = 43; P < 0.001^b^)

^a^ significant difference within a group.

^b^ significant difference between groups.

Abbreviations: 95%CI = 95% confidence interval; ES = effect size.

## 4 Discussion

This systematic review and meta-analysis compare the overall impact of SSG and HIIT players’ physical fitness. In general, both training modalities substantially enhance athletic performance; however, they promote distinct physiological adaptations and target different fitness components, thereby offering valuable insights for optimising training protocols.

Despite their overall effectiveness, the improvements in power were relatively modest (ES = −0.144, p > 0.05), suggesting inherent limitations in the capacity of SSG and HIIT interventions to maximise muscular force output fully. From a physiological standpoint, the development of power typically requires targeted, high-load resistance training (Morris et al., 2022). While HIIT can improve neuromuscular coordination through brief bursts of explosive effort, its stimulus may prove insufficient for significant power gains; similarly, SSG, which predominantly emphasises multidirectional movements and tactical decision-making within game-like contexts, appears to exert a comparatively weaker direct effect on power enhancement.

The meta-analysis further demonstrates that both SSG and HIIT significantly improve speed (ES = 0.564) and endurance (ES = 0.706), primarily attributable to their positive influence on cardiorespiratory function and neuromuscular adaptation. HIIT, through repeated high-intensity sprints, effectively activates the anaerobic energy system, thereby increasing phosphocreatine reserves and lactate tolerance, which confers an advantage in acceleration and short-term explosive performance (Boullosa et al., 2022). In contrast, SSG, by emulating real-game conditions, fosters continuous movement and rapid change-of-direction, thereby enhancing aerobic endurance and recovery capacity (Merks et al., 2022). The low heterogeneity (I^2^ = 0%) underscores the consistency of these effects across studies.

Although the improvements in agility (ES = −0.159) did not achieve statistical significance, the observed trend indicates that both interventions contribute to enhanced quick directional changes and coordination. The game-like demands of SSG necessitate swift decision-making in dynamic environments, thereby refining multi-joint coordination and movement adaptability (Bujalance-Moreno et al., 2022), whereas HIIT primarily focuses on high-intensity output and recovery, potentially offering less short-term stimulation for agility. The complementary nature of these two modalities intimates that an integrated training approach may yield superior overall fitness enhancement.

Subgroup analysis reveals a nuanced influence of factors such as training frequency, intervention duration, age, and gender on training outcomes. Notably, interventions conducted fewer than three times per week or lasting less than 6 weeks produced significant improvements; however, increasing the frequency or extending the duration beyond this threshold resulted in diminishing returns, highlighting the critical balance between training stimulus and recovery. Both novice and experienced players benefit from high-intensity training, although mature players (aged ≥16) may exhibit more pronounced neuromuscular adaptations and power gains. While single-sex samples did not yield statistically significant outcomes, studies incorporating mixed-gender participants demonstrated moderate effects, suggesting that gender may not be a primary moderating factor, though the limited representation of female players necessitates cautious interpretation.

## 5 Limitations

There are several limitations to this system review. Firstly, this review only includes English articles sourced from PubMed, Web of Science, SCOPUS, and EBSCOhost databases, potentially overlooking relevant publications from other sources. Secondly, a majority of studies lack precise delineation of intervention durations, precluding analysis of intervention duration within this review. Thirdly, this review does not encompass other sports pertinent to jumping performance, such as badminton, volleyball, and gymnastics. Fourthly, owing to a limited number of studies, this systematic review only compares HII against small-sided games, omitting comparisons with alternative training modalities like plyometric training and core training. Fifthly, the binary approach employed in moderating variable analysis, involving median segmentation of continuous data, may engender residual ambiguity and diminish statistical robustness. Lastly, given the scarcity of studies focused specifically on females, the applicability of our meta-analysis results to the female population may4 be limited.

## 6 Conclusion

This systematic review and meta-analysis demonstrate that both SSG and HIIT are effective strategies for enhancing players’ physical fitness, with significant improvements observed in speed and endurance. While the enhancements in power and agility are more modest, the distinct physiological adaptations of each modality underscore their potential for targeted training interventions. The evidence supports the integration of HIIT and SSG into comprehensive training programs tailored to the specific demands of various sports and athlete populations. Continued research is essential to refine these protocols and fully exploit the benefits of high-intensity training strategies. Future research should focus on investigating the long-term effects of HIIT and SSG on athletic performance and determining the sustainability of the observed adaptations. Additionally, optimising training protocols by exploring the ideal balance between training frequency and intervention duration will be crucial.

## 7 Contributions to the literature

Systematic comparative evidence regarding the effects of High-Intensity Interval Training (HIIT) and Small-Sided Games (SSG) remains limited, particularly within athletic populations.

This study identifies differences between the two training modalities in key physical fitness components such as endurance and speed, thereby providing quantitative evidence to inform sports training practice.

The findings suggest that coaches and practitioners may select more targeted training methods based on athletes’ specific performance goals.

Future research should further investigate the long-term intervention effects of HIIT and SSG under standardised measurement indicators.

## Data Availability

The original contributions presented in the study are included in the article/supplementary material, further inquiries can be directed to the corresponding authors.
